# Refining the Performance of Routine Information System Management (PRISM) framework for data use at the local level: An integrative review

**DOI:** 10.1371/journal.pone.0287635

**Published:** 2023-06-27

**Authors:** Nami Kawakyu, Megan Coe, Bradley H. Wagenaar, Kenneth Sherr, Sarah Gimbel

**Affiliations:** 1 Department of Global Health, University of Washington, Seattle, Washington, United States of America; 2 Department of Child, Family, & Population Health Nursing, University of Washington, Seattle, Washington, United States of America; 3 Department of Epidemiology, University of Washington, Seattle, Washington, United States of America; 4 Department of Industrial and Systems Engineering, University of Washington, Seattle, Washington, United States of America; South African Medical Research Council (SAMRC) / Stellenbosch University (SU), SOUTH AFRICA

## Abstract

**Introduction:**

Foundational to a well-functioning health system is a strong routine health information system (RHIS) that informs decisions and actions at all levels of the health system. In the context of decentralization across low- and middle-income countries, RHIS has the promise of supporting sub-national health staff to take data-informed actions to improve health system performance. However, there is wide variation in how “RHIS data use” is defined and measured in the literature, impeding the development and evaluation of interventions that effectively promote RHIS data use.

**Methods:**

An integrative review methodology was used to: (1) synthesize the state of the literature on how RHIS data use in low- and middle-income countries is conceptualized and measured; (2) propose a refined RHIS data use framework and develop a common definition for RHIS data use; and (3) propose improved approaches to measure RHIS data use. Four electronic databases were searched for peer-reviewed articles published between 2009 and 2021 investigating RHIS data use.

**Results:**

A total of 45 articles, including 24 articles measuring RHIS data use, met the inclusion criteria. Less than half of included articles (42%) explicitly defined RHIS data use. There were differences across the literature whether RHIS data tasks such as data analysis preceded or were a part of RHIS data use; there was broad consensus that data-informed decisions and actions were essential steps within the RHIS data use process. Based on the synthesis, the Performance of Routine Information System Management (PRISM) framework was refined to specify the steps of the RHIS data use process.

**Conclusion:**

Conceptualizing RHIS data use as a process that includes data-informed actions emphasizes the importance of actions in improving health system performance. Future studies and implementation strategies should be designed with consideration for the different support needs for each step of the RHIS data use process.

## Introduction

At the foundation of a well-functioning health system is a health information system that informs decisions and actions regarding health system financing, leadership and governance, health workforce, service delivery, and essential medicines [1]. Routine health information systems (RHISs) are implemented at scale in low- and middle-income countries (LMICs) and inform decision making at all levels of the health system [2–4]. RHISs are characterized by systems that ensure the production, analysis, and use of data at intervals of less than a year, to generate information regarding health system performance such as quantity of health services delivered [5,6].

Widespread adoption and implementation of health system decentralization in LMICs has meant that decision making responsibilities to manage health services has shifted to officials at the local levels, particularly to facility and district or equivalent administrative levels [7,8]. Decentralization is theorized to contribute to improved primary health service provision, by allowing local officials to manage service delivery to be more responsive to local needs [7,8]. Some have conceptualized a synergistic relationship between RHIS and decentralization, in that decentralization encourages local health system managers to use RHIS data to make decisions and take action, while the availability of RHIS data generated at the health facility enables a decentralized health system [8–12]. There is also evidence that when frontline health workers, who are typically responsible for collecting RHIS data, are engaged in using the data themselves to inform clinical practice and service delivery, they come to see the value of the data, leading to improvements in data quality and data-informed decision making [11,13–16].

However, RHIS data use remains low across many LMICs, with many countries collecting large amounts of data but never analyzing and reviewing it to inform decisions and actions to improve health system performance [6,12,14,16–21]. Many health facilities also suffer from “mailbox syndrome,” in which RHIS data is sent to higher administrative levels for analysis or reporting, without use at the facility to inform improvements [21–24]. Additionally, recent systematic reviews have found that many RHIS data use interventions focus on strengthening the data collection and analysis skills of health workers [5,6,15,25,26], but do not focus on the critical step of translating data into action [15,17,27], which is what is ultimately needed to improve health system performance and population health. A leading RHIS data use expert called out this gap in a 2017 editorial:

*“Most efforts to strengthen health facility and community health information systems are focused on… identifying problems. But the ultimate goal of RHIS is that information is used to solve problems and to improve access to and delivery of quality health services. This last step of translating data into action is the most challenging” [27]*.

This highlights that “RHIS data use” is a multi-step process, whereby identifying problems and taking action are crucial and represent distinct steps within the “RHIS data use” process. However, RHIS data use papers often do not detail what steps are included within the “RHIS data use” process.

In the Performance of Routine Information System Management (PRISM) framework [9], a popular framework used to evaluate and inform the development of RHIS strengthening interventions in LMICs, there is a direct pathway from improved RHIS data quality and use to improved health system performance, but it is unclear within the framework whether, where, or how data is translated into action. Accordingly, some studies using the PRISM framework have called for a need for more clarity in conceptualizing the complex relationship between RHIS data, data use, decision making, and health system impact [5,16,28].

Relatedly, systematic reviews have noted the lack of consensus and consistency in how RHIS data use is conceptualized and measured [15,28], impeding the development, evaluation, and identification of effective RHIS data use interventions. To date, there has not been a comprehensive review of how “RHIS data use” is conceptualized and measured in the literature.

The purpose of this integrative review was to synthesize published literature on how “RHIS data use” at the district and health facility-levels in LMIC settings is conceptualized and measured, in order to inform the refinement of the PRISM framework, identify a common definition for RHIS data use, and propose improved measurement approaches to move the RHIS data use field forward.

## Methods

An integrative review methodology was used to summarize the current state of the literature and take a comprehensive approach to fully understand the concept of “RHIS data use,” including identifying data collection tools and analytic methods used to measure RHIS data use. Integrative review is a type of systematic review method to summarize literature of various methodologies to provide a more comprehensive understanding of a particular phenomenon [29]. The process of systematically analyzing, visualizing, and comparing data from literature enables the identification of patterns and themes to generate new or revised concepts and frameworks [30,31]. The Preferred Reporting Items for Systematic Reviews and Meta-Analysis (PRISMA) 2020 guidelines were followed in the reporting of this review [32] (S1 File).

Given the wide use of the PRISM framework and its associated data collection tools, we selected the PRISM framework for refinement rather than developing a new conceptual framework. The PRISM framework aligned with the aim of this review as it is the only health information system framework developed specifically for RHISs in the LMIC context [5,9,14].

### Eligibility criteria

Studies included in this review were peer-reviewed research papers published in English investigating RHIS data use at the district or health facility level in LMIC settings (S1 Table). For the purposes of the search and selection process, RHIS data use was defined broadly as any consideration of RHIS data to inform health system management decisions or actions. Studies were excluded if RHIS data use was not part of the main findings but instead discussed in the background or discussion sections only. Articles reporting results from RHIS implementation or data quality assurance activities without directly investigating and reporting on RHIS data use were excluded. Literature review and synthesis papers, as well as studies using quantitative, qualitative, or mixed methods designs were included. Given that the PRISM framework was published in 2009 and the rapid advancements in this field, searches were limited to those published between 2009 and 2021, to capture the most current understanding and practices on this topic.

### Information sources and search strategy

The following databases were systematically searched in January 2022 to identify potential papers: EBSCO CINAHL, EBSCO Global Health, Embase, and PubMed. A combination of search terms was used to identify published studies (S2 Table). Databases and search terms were selected based on the primary purpose of the review and previous reviews of similar topics [5,6,26,33]. Reference lists of included systematic review articles were also searched to identify additional publications.

### Selection process

All article records identified through the database search were exported into Excel; articles identified through the reference list search were entered into the same spreadsheet. Upon identification and removal of duplicate records, titles and abstracts of remaining articles were screened and classified by two reviewers as “eligible” or “ineligible,” citing the relevant eligibility criteria. Full-text articles of all articles identified by at least one reviewer as eligible were then reviewed and further classified as eligible or ineligible by both reviewers, noting reasons for exclusion. Any disagreements in selection were discussed and resolved.

### Data collection process, data items, and synthesis

#### Data use concept: Terminology, definitions, application, and steps

All included articles were read and coded by an analyst in ATLAS.ti (v8) using the initial codes “terms,” and “definition.” To systematically apply codes, a codebook was developed with code definitions, inclusion and exclusion criteria, and examples. Upon completion of initial coding, the ATLAS.ti search term and Word List functions were used to identify and code any additional data use terminology (i.e., data use synonyms) used across all included articles. Coding reports were exported into Excel for synthesis.

To synthesize the terminology data, similar data use terms were combined to create umbrella terms. For example, “use” and “utilization” were combined to be “data use/utilization.” Differences in ordering of terms were also merged into one term, for example, “use-of-information” was categorized under “information use/utilization.” Summary statistics were calculated to assess which words were most used across articles included in this review.

Summary statistics were calculated to assess what percentage of articles explicitly defined data use. Thematic analysis of data use definitions was conducted to group similar definitions. Through this process, the themes of “application” and “steps,” emerged, in that some articles defined data use by its application and others by its steps. All articles were then reviewed to apply the codes “application,” in other words, for what purposes data is used such as for planning and monitoring purposes, and “steps,” which was inclusive of any RHIS data tasks such as data collection, data analysis, and decision making based on data. “Application” and “steps” were defined to be mutually exclusive; “applications” are the reasons for data use, while “steps” were physical actions taken. For example, health facility staff may hold a meeting to discuss and make a decision based on analyzed data (“steps”), for the purpose of budgeting (“application”). Coding reports were then exported into Excel for synthesis.

Application data was used to create a full list of data use applications. Similar applications were combined to create umbrella applications. For example, “budgeting” and “resource allocation,” were grouped into one umbrella “budgeting/resource allocation” application. Each article was then reviewed to determine which data use applications were noted and then summary statistics were calculated to assess which applications were most reported across included articles by distinct first authors.

To synthesize the data use steps data, the ATLAS.ti coding report informed the development of a comprehensive list of RHIS data tasks. Extracted text were then assessed to fall under these RHIS data tasks and color coded based on whether the authors implied the task to be a separate or inclusive part of the “RHIS data use” process. For example, if the extracted text said, “data analysis, interpretation, and use,” we specified that “data analysis,” and “interpretation,” were RHIS data tasks that were not a part of “data use,” whereas if the extracted text said, “The ‘use’ of data is the analysis, synthesis, interpretation, and review of data as part of a decision-making process,” [30] then we noted that data analysis, synthesis, interpretation, review, and decision making were inclusive of the data use process. The number of articles that considered each RHIS data task as part of, or not a part of, data use was counted. Each RHIS data use task was then categorized as:

1. Consensus, not data use (all authors agree that task is not a part of data use),
2. Some disagreement, leans not data use (most authors agree that task is not a part of data use),
3. Some disagreement, leans data use (most authors agree that task is part of data use), and
4. Consensus, data use (all authors agree that task is a part of data use).

#### Data use measurement

A “data use measurement” data collection tool was designed, tested, and refined by the investigators to extract data from articles on approaches to measuring RHIS data use. Design of the items in the tool were guided by measurement sciences principles [34–37] and included:

- Author, publication year, title, and country,
- Study design,
- Data collection tool used (e.g., questionnaire) and its source (e.g., PRISM toolkit),
- Number of items in the tool,
- Items in the tool (e.g., the actual questions and response options such as Yes/No), and
- How the data use measure was calculated.

Risk of bias and certainty of evidence assessments were not conducted because the focus of the review was methodological and not concerned with conducting statistical analyses of the study results.

### Framework refinement

The integrated review informed the development of a refined framework, with the primary aim of the refined framework and definitions to clarify, “What is RHIS data use? What does RHIS data use look like?”

The most commonly used term for “RHIS data use” across the literature was selected for the refined framework. RHIS data tasks identified through the synthesis were merged with tasks in the PRISM framework. Tasks assessed to be a part of, or not a part of, the data use process based on author agreement, were placed accordingly in the framework. For tasks with some disagreement, we reviewed relevant article texts to discuss and determine appropriate task placement in the framework. The same process of article text review and group discussion was taken to determine the appropriate groups of RHIS data use tasks such as data capturing, data processing, and data dissemination. Once activities under “RHIS data use” were determined, article texts informed the definitions for each step. Once definitions were drafted, they were reviewed against the refined framework to assess consistency across the framework and definitions.

## Results

### Study selection

**Fig 1:**
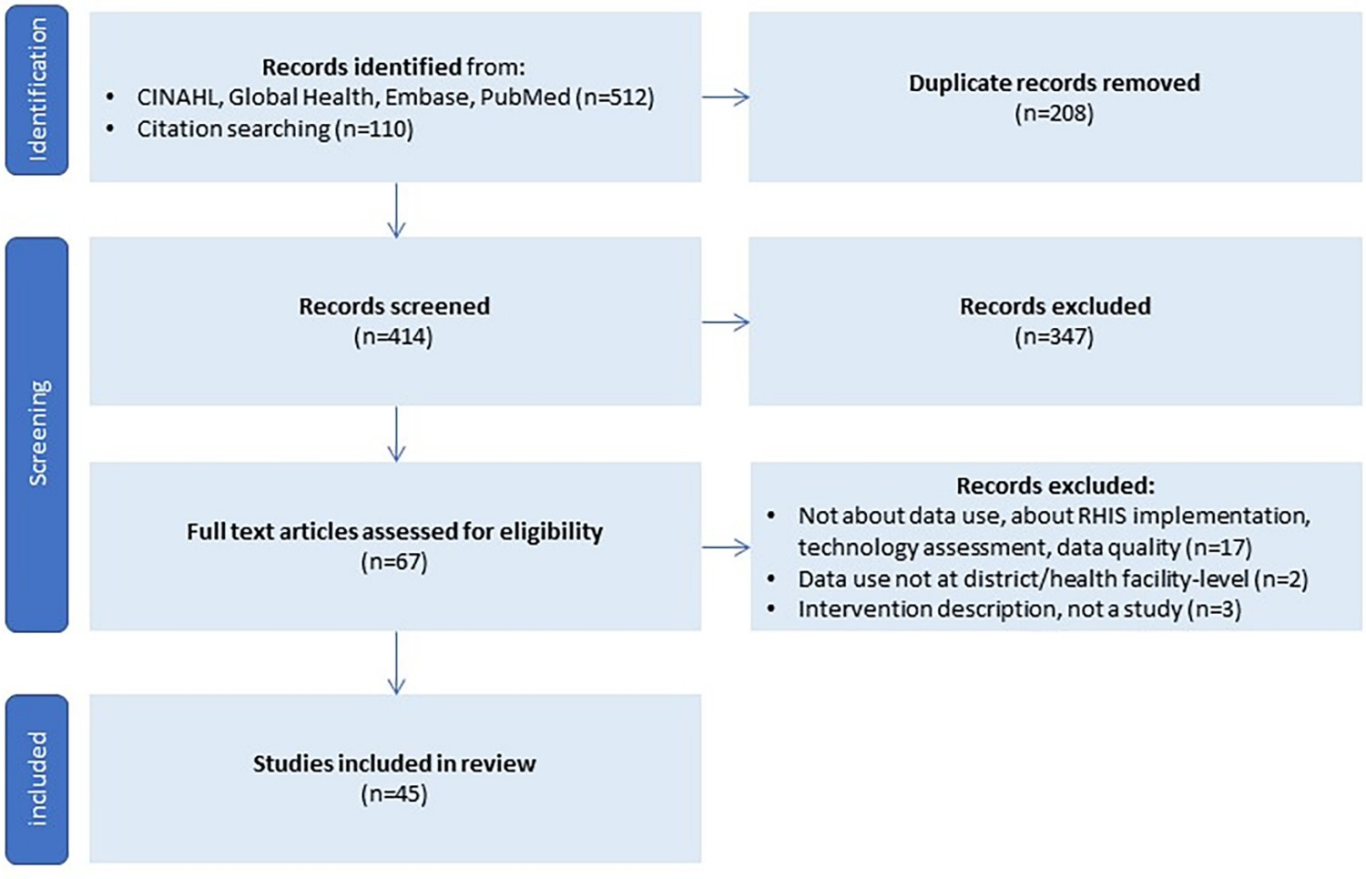
Study selection process.

A total of 622 articles were identified: 512 through the database searches and an additional 110 through citation searching. After removing duplicates, 414 articles remained and were screened based on their title and abstract. Of these, 347 were excluded, primarily because they were off-topic or outside the geographic scope of the study. 67 full-text articles were assessed for eligibility, of which 17 were excluded because they focused on other aspects of RHIS such as implementation and data quality. A total of 45 articles met the inclusion criteria. Fig 1 summarizes the study identification, screening, and selection process.

### Study characteristics

There was a nearly even distribution of study designs used by the papers included in the review (Table 1). A few studies reported to use mixed-methods for the overall study but only used qualitative methods to assess data use; these were classified as qualitative studies. Among the 24 articles using quantitative methods, 10 articles (42%) used descriptive analysis to report data use as a count and/or percentage and did not conduct any inferential analysis. 10 articles (42%) conducted tests of association to assess what determinants were associated with data use, three used a pre/post design to assess change over time, and one tested the reliability and validity of the PRISM data collection tool. The median publication year was 2020, with more than half of the articles (53%) published between 2020 and 2021.

**Table 1:** Study characteristics.

Characteristics	n (%), unless noted
*Study design (N = 45)* Qualitative Quantitative Mixed methods Synthesis	9 (20%) 12 (27%) 12 (27%) 12 (27%)
*Published year*, *median (range)*	2020 (2009–2021)
*Regions (N = 33*^*a*^*)* Ethiopia Central and West Africa Eastern and Southern Africa Asia Multiple regions	12 (36%) 5 (15%) 7 (21%) 7 (21%) 2 (6%)

^a^ Original research articles only, excludes synthesis articles.

The vast majority of the 33 original research articles were conducted in sub-Saharan Africa (73%), with studies conducted in Ethiopia accounting for more than a third of all original research articles (36%).

### What is meant by “RHIS data use”?

#### Terminology

A total of 17 terms for “RHIS data use” were identified (Table 2). The most commonly used terms across the papers included in the review were “data use” (89%), “information use” (71%), “data use for/in decision making” (58%), and “evidence-based/informed decisions” (51%). “Decisions” and “decision making” were common additions to the terms “data use” and “information use.”

**Table 2:** Number (%) of articles using specified data use terms (N = 45).

Data use terms	n (%)
Data use Information use Data use for/in decision making Evidence-based/informed decisions Data-based/driven/informed decisions Information use for/in decision making Information use for evidence-based decisions Other	40 (89%) 32 (71%) 26 (58%) 23 (51%) 15 (33%) 12 (27%) 8 (18%) 8 (18%)

#### Definition

Among the 45 articles reviewed, less than half (42%) explicitly defined “RHIS data use.” While there was some commonality across data use definitions (Table 3), there was not a commonly accepted and cited definition for “RHIS data use.” Nearly half of the definitions (47%) focused on the application of RHIS data use, while about a quarter (26%) focused on the steps involved in RHIS data use. The most common word used when defining data use was “decision.” A few articles explicitly defined decision making.

**Table 3:** Data use and decision making definitions reported by articles.

Article	Country	Definition
Data use is defined by decisions and actions informed by data		
Abajebel 2011 [22] Bogale 2021 [38]	Ethiopia	“Using information for decision making to take immediate action.”
Lazzerini 2019 [39]	Sri Lanka	“Any action-oriented recommendation generated from review of the data outcomes.”
Lemma 2020 [26]	Synthesis	“Use of routine health information system data for decision making.”
Leon 2020 [28]	Synthesis	“The capacity and processes for effective decision making”
**Data use is defined by its application, i.e., the types of decisions made based on the data**		
Hotchkiss 2012 [5]	Synthesis	“Decision makers explicitly considering information in policymaking, planning, management, and service delivery.”
Nutley 2014 [16]	Côte d’Ivoire	“‘Data-informed decision making’ refers to the proactive and interactive processes that consider data during program monitoring, review, planning, and improvement; advocacy; and policy development and review.”
Shiferaw 2017 [40]; Dagnew 2018 [41]; Wude 2020 [42]	Ethiopia	Use of routine health information to: • Monitor, manage, and improve day-to-day health service activities • Monitor and manage drug procurement and supply • Display and share updated information of key indicators • Predict, identify, and manage epidemics/outbreaks • Identify and prioritize community health problems • Mobilize the community • Mobilize and allocate resources • Check data quality • Evaluate department and staff performance
Rendell 2020 [33]	Synthesis	“The concept of leveraging data or findings from analyses of a set of indicators to improve health system performance.”
Kumar 2021 [43]	Synthesis	“The analysis, synthesis, interpretation and review of data as part of decision-making processes such as program monitoring, policy development, and resource allocation.” “‘Data to action’ refers to data-based decision making that shows a measurable impact of improvements in processes, systems, human resources, and institutional attributes.”
Seid 2021 [44]	Ethiopia	“Use of health information in decision making, such as for planning, monitoring, evaluation, treating patients/services, disease prioritization, budget allocation, supervision, writing feedback, showing trends, and quality data reporting.”
Tulu 2021 [45]	Ethiopia	“Using routine health information for service improvement, patient treatment, staff performance, planning, department evaluation, monitoring key performance indicators, prediction of outbreaks, resource allocation, development of policy and advocacy.”
**Data use is defined by its processes, i.e., the completion of specific tasks**		
Aqil 2009 [9]	Synthesis	“Use of information for identifying problems, for considering or making decisions among alternatives, and for advocacy.”
Nutley 2013 [14]	Synthesis	“The ‘use’ of data is the analysis, synthesis, interpretation, and review of data as part of a decision-making processes.”
Endriyas 2020 [17]	Ethiopia	The process which encompasses, “problem identification, problem prioritization, preparing an action plan, monitoring implementation of action plan, data visualization, and assessing data quality.”
Chanyalew 2021 [46]	Ethiopia	“The process that encompasses problem identification, prioritization, action plan development, implementation, and following-up, and providing regular feedback.”
Osterman 2021 [15]	Synthesis	“A process in which data collected by the health system are converted into usable information through data processing, analysis, synthesis, interpretation, review, and discussion, then used to decide on a course of action.”
**Decision making definitions**		
Wickremasinghe 2016 [12]	Synthesis	“The process by which a group of people reach a collective understanding of a topic, which then helps to build consensus on a particular course of action to address a health service challenge, from two or more possible options.”
Bhattacharyya 2020 [47]	India	“Stakeholders reaching consensus on a particular course of action from two or more possible options to address health service challenges.”
Tulu 2021 [45]	Ethiopia	“The process of identifying and choosing alternatives based on the values, preferences, and beliefs of the decision-maker.”

#### Application

Among the 45 articles reviewed, most (84%) described the reasons RHIS data was used by districts and health facilities. There was great variability across articles in who the specified data users were, which included community health workers, health workers, health information and monitoring and evaluation staff, as well as facility and district managers. Data use applications noted by the greatest proportion of papers with distinct first authors were improvement of program/service delivery (76%), planning (71%), and performance monitoring and evaluation (68%) (Table 4). General planning, prioritization, and management were commonly mentioned and were kept separate from specified management activities such as human resource management and drug/commodities management to reflect how these were presented in the papers.

**Table 4:** Number (%) of articles specifying data use applications (N = 38).

Data use applications	n (%)
Service delivery/program improvement Planning Performance monitoring and evaluation Policy/strategy development Budget/resource allocation Management Human resource management Drug/commodity management Prioritization Patient clinical care Disease detection and prioritization Advocacy Reporting Data quality assessment	29 (76%) 27 (71%) 26 (68%) 19 (50%) 19 (50%) 17 (45%) 10 (26%) 9 (24%) 8 (21%) 8 (21%) 8 (21%) 7 (18%) 7 (18%) 2 (5%)

**Steps.** Among the 45 articles reviewed, very few (11%) explicitly described the steps that take place when RHIS data is “used,” i.e., answering “What does data use look like?” One study centered their definition of data use around the steps included in the data use process, which were “problem identification, prioritization, action plan development, implementation, and following-up, and providing regular feedback” [46].

Most studies (64%) noted various RHIS data-related tasks; 16 distinct tasks were identified. Among these, 25 articles and 21 distinct first authors implied these tasks to be a separate or inclusive part of the RHIS data use process. There was consensus that RHIS data collection and recording, transmission, and processing were not a part of data use and that problem identification, prioritization, solving, decision making, action planning, action taking, and monitoring/follow-up were a part of data use (Table 5). There was some disagreement about the other RHIS data tasks but generally, data quality assessments, data analysis, dissemination of data (through presentation or display of data visualizations), reporting, and data interpretation were not considered to be a part of the data use process. Review and discussions about data were generally considered to be a part of data use.

**Table 5:** Classification of consensus regarding RHIS data tasks (N = 25).

RHIS data tasks	Part of data use?		
	No	Yes	Legend
Collection/recording	11	0	Consensus, not data use
Transmission	2	0	Some disagreement, leans not data use
Processing	7	0	Some disagreement, leans data use
Data quality assessment	3	1	Consensus, data use
Data analysis	13	3	
Dissemination (presentation, display)	6	2	
Reporting	10	2	
Interpretation	8	3	
Review	2	4	
Discussion	1	4	
Problem identification	0	2	
Problem prioritization	0	2	
Problem solving/decision making	0	6	
Action planning	0	3	
Action	0	2	
Monitoring/follow-up	0	3	

### How is RHIS data use measured?

Among the 24 studies measuring RHIS data use, more than half (54%) used an adapted version of the PRISM toolkit (Table 6). The PRISM toolkit is a suite of six different data collection tools to aid in the assessment of RHIS performance [48]. In particular, the RHIS Performance Diagnostic Tool collects data to measure data use at the district-level as well as at the facility-level. The toolkit was initially developed by MEASURE Evaluation in 2011 [49] and then updated by MEASURE in 2019 [48]. The 2011 RHIS Performance Diagnostic Tool at the facility-level contains 26 items mostly with Yes/No response options, compared to the 2019 tool with 32 items with Yes/No response options as well as categorical, numeric, and open-ended response options. A study assessing the 2011 PRISM toolkit found evidence to suggest that the tools are reliable and valid but noted that there was limited data availability to measure “RHIS data use” and that findings should be interpreted with caution [50]. To our knowledge, there have not been any papers published on the reliability and validity of the 2019 PRISM toolkit.

**Table 6:** Approaches to measuring RHIS data use (N = 24).

Data collection tool, source	n (%)
PRISM (adapted)	13 (54%)
Developed by study	8 (33%)
Other	2 (8%)
Not reported	1 (4%)
**Data collection tool, type**	
Questionnaire only	8 (33%)
Observation/document review tool only	7 (29%)
Both (questionnaire + observation)	7 (29%)
Program data	1 (4%)
Not reported	1 (4%)
**Data collection tool, number of items**	
1–5	6 (25%)
6–10	5 (21%)
11+	6 (25%)
Not reported	7 (29%)
**Data use measure**	
Binary	11 (46%)
Categorical	1 (4%)
Continuous	2 (8%)
Not reported/calculated	10 (42%)

Among the 13 studies using an adapted version of the PRISM toolkit, there was wide variation in the approach to measuring data use. For example, one study using the PRISM toolkit only used one item to measure data use [50], while another study using the toolkit measured data use through 13 questionnaire items and 8 observation tool items [41]. Though the PRISM framework clearly delineates “data analysis” and “data display” as part of RHIS processes that precede data use, a quarter of the studies (25%) included data analysis and/or data display measures as part of their “data use” measure. It was not possible to assess this for nearly a third of the studies (29%) because they did not report what items they collected to measure data use.

Most studies used a questionnaire (33%), observation or document review (29%), or both (29%) to measure data use. The exception was a study that developed a decision tracking system which served as their data source for the number and types of decisions taken and actions planned and completed [51]. About a third of studies (33%) collected self-reported measures about data use from health facility and/or district-level staff, without verification through observations or document review.

The number of items used to measure data use varied widely, ranging from one to 33 items, with a median of eight items. Nearly half of the studies (42%) either did not calculate or report an overall “data use” measure, instead reporting on the percentage of health facilities that said yes or no to individual tool items. Studies testing the association of “data use” with data use determinants commonly transformed the data use measure into a binary variable (e.g., good/poor data use). Mean or median values were commonly used as the cut-off for defining binary variables. A summary of measurement approaches of reviewed studies is available in Table 7.

**Table 7:** Data use measurement approaches reported by articles.

Country	Author, Year	Data collection tool	Data use items in tool (Response options Yes/No unless specified)	Data use measure
Ethiopia	Abajebel 2011 [22]	Study-designed questionnaire and observation tool	1. Received feedback from supervisor 2. Calculated area coverage and prepared maps 3. Presented key indicators with charts or tables 4. Presented achievement of targets	• Data use: Yes to 3–4 items • No data use: Yes to 0–2 items
Ethiopia	Shiferaw 2017 [40]	PRISM (adapted) questionnaire and observation tool	5-point agreement Likert scale applied to: 1. Uses data for day-to-day management of health service facilities and districts 2. Displays data for monitoring the key objectives of health services and showing key indicators by means of graphs and tables 3. Finds out whether the health professional can gather data to detect the cause of health problems to prioritize the problems and use the data for health education 4. Uses data to identify and manage epidemics 5. Uses data to observe trends of health services and for supply and management	• Good data use practice: Above the mean • Poor data use practice: Equal to or below the mean
Ethiopia	Dagnew 2018 [41]	PRISM (adapted) questionnaire and observation tool	• 13 items in questionnaire (5-point agreement Likert scale) • 8 items in observation tool	• Good data use: Above the mean • Poor data use: Equal to or below the mean
Ethiopia	Endriyas 2020 [17]	Study-designed document review tool (based on MEASURE 2009 [52])	1. Availability of performance review minutes 2. Minutes address high and low performance 3. Action plan indicates problem prioritization 4. Activities report indicate actions taken 5. Availability of up-to-date monitoring charts 6. Evidence of assessment of data quality	• Good data use: 75%+ • Fair data use: 50–74% • Poor data use: <50%
Ethiopia	Kebede 2020 [53]	PRISM (adapted) questionnaire	Not reported.	Binary; calculation method not reported.
Ethiopia	Wude 2020 [42]	PRISM (adapted) questionnaire and Federal Ministry of Health (adapted) document review tool	• 10 items in questionnaire (5-point frequency Likert scale) • 4 items in document review tool	Good/poor data use: cut-off not reported.
Ethiopia	Bogale 2021 [38]	PRISM (adapted) questionnaire and observation tool	Not reported in methods section. Reports the following in results section: 1. Monthly performance review meetings and monthly data analysis 2. Use data for decision making 3. Display achievement of target, population profile, and staffing by table or graph or chart in the service delivery unit	Not reported.
Ethiopia	Chanyalew 2021 [46]	PRISM (adapted) questionnaire	1. Presence of feedback from supervisor (response option not reported) 2. Evidence on the use of information for decision making (response option not reported)	• Good data use: Above the mean • Poor data use: Equal to or below the mean
Ethiopia	Gonete 2021 [54]	Document review tool (source not reported)	Not reported.	Not reported.
Ethiopia	Seid 2021 [44]	PRISM (adapted) questionnaire	Data used for: 1. Monitoring day-to-day activities 2. Treating patients/provide service 3. Prioritizing problem 4. Showing a key performance by the chart 5. Performance evaluation 6. Observing trends of service 7. Planning 8. Reporting of quality data 9. Taking action 10. Information dissemination	• Continuous % variable for descriptive analysis • Good data use: Mean score ≥65% • Poor data use: Mean score <65%
Ethiopia	Tulu 2021 [45]	PRISM (adapted) questionnaire	Data used for: 1. Service improvement 2. Patient treatment 3. Staff performance 4. Planning 5. Department evaluation 6. Monitoring key performance indicators 7. Prediction of outbreaks 8. Resource allocation 9. Development of policy 10. Advocacy	• Uses data: ≥5 yeses • Does not use data: ≤4 yeses
Tanzania	Nyamtema 2010 [24]	Study-designed questionnaire	Not reported. Tool available as appendix, appears to have one question: • For what purposes do consumers of data utilize the data: policy making, planning and budgeting, evaluation of health programs, other?	Does not report method to calculate overall data use measure. Reports % of “no” item responses.
Tanzania	Mboera 2021 [19]	Study-designed questionnaire and observation tool	Not reported.	Not reported.
Uganda	Hotchkiss 2010 [50]	PRISM (adapted) questionnaire and observation tool	2004 and 2007: 1. Display of a map, chart, or table based on RHIS data 2007 only: 2. RHIS information discussed in staff meetings 3. RHIS information used to make decisions 4. RHIS information used to take follow-up actions or refer for action 5. Display of a map, chart, or table based on RHIS data	• Use of RHIS information: Yes to item 1 (2004) • Continuous composite measure (2007)
South Africa	Kawonga 2013 [55]	Study-designed questionnaire	1. Reads HIV data report for own level 2. Discusses HIV data with managers at own level 3. Discusses HIV data with managers at lower level/own facility staff 4. Discusses HIV data with managers at higher level 5. Interprets data: monitors against targets 6. Interprets data: compares to previous time periods 7. Makes decisions/takes action based on the data/indicator levels	Binary; calculation method not reported.
South Africa	Nicol 2017 [23]	PRISM (adapted) document review tool	1. RHIS report production 2. Frequency of RHIS reports 3. Types of reports produced 4. Display of information at the facility-level 5. Use of information in available reports at facility 6. Types of decisions based on types of analyses 7. Discussion and decisions based on RHIS information 8. Promotion and supervision by the district office	Does not report method to calculate overall data use measure. Reports % of “yes” item responses.
Cote d’Ivoire	Nutley 2014 [16]	PRISM tool (adapted); specifics not reported	1. RHIS information was discussed in staff meetings 2. Decisions evolved from these discussions 3. Decisions were referred to upper management for action	Continuous composite measure (calculation specifics not reported).
Nigeria	Nwankwo 2018 [56]	Study-designed document review tool	Not reported.	Not reported.
Ghana	Odei-Lartey 2020 [57]	PRISM (adapted) document review tool	15 items in document review tool	Does not calculate overall data use measure. Reports % of “yes” item responses.
Cameroon	Nguefack-Tsague 2020 [58]	MEASURE Data Analysis, Dissemination, and Use tool [59]	18 items in document review tool (response options not reported)	Calculates and reports global RHIS performance score: • Good score: ≥60% • Poor score: <60%
Cameroon	Tamfon 2020 [60]	MEASURE Data Analysis, Dissemination, and Use tool [59]	18 items in document review tool with response options: 0 = no answer/not applicable 1 = not present, needs to be developed 2 = needs a lot of strengthening 3 = needs some strengthening 4 = already present, no action needed	Does not report method to calculate overall data use measure. Reports % of select response items.
India	Prakash 2021 [51]	Study-designed decision tracking system	1. Number of decisions taken 2. Number of actions completed	Frequency calculated for a continuous data use scale.
Pakistan	Nawaz 2020 [61]	PRISM (adapted) questionnaire	33 items in questionnaire	Does not calculate overall data use measure. Reports % of response items.
Pakistan	Kumar R 2012 [62]	Study-designed questionnaire	Not reported.	Not calculated.

### Refining the PRISM framework: The PRISM-Act framework

**Fig 2:**
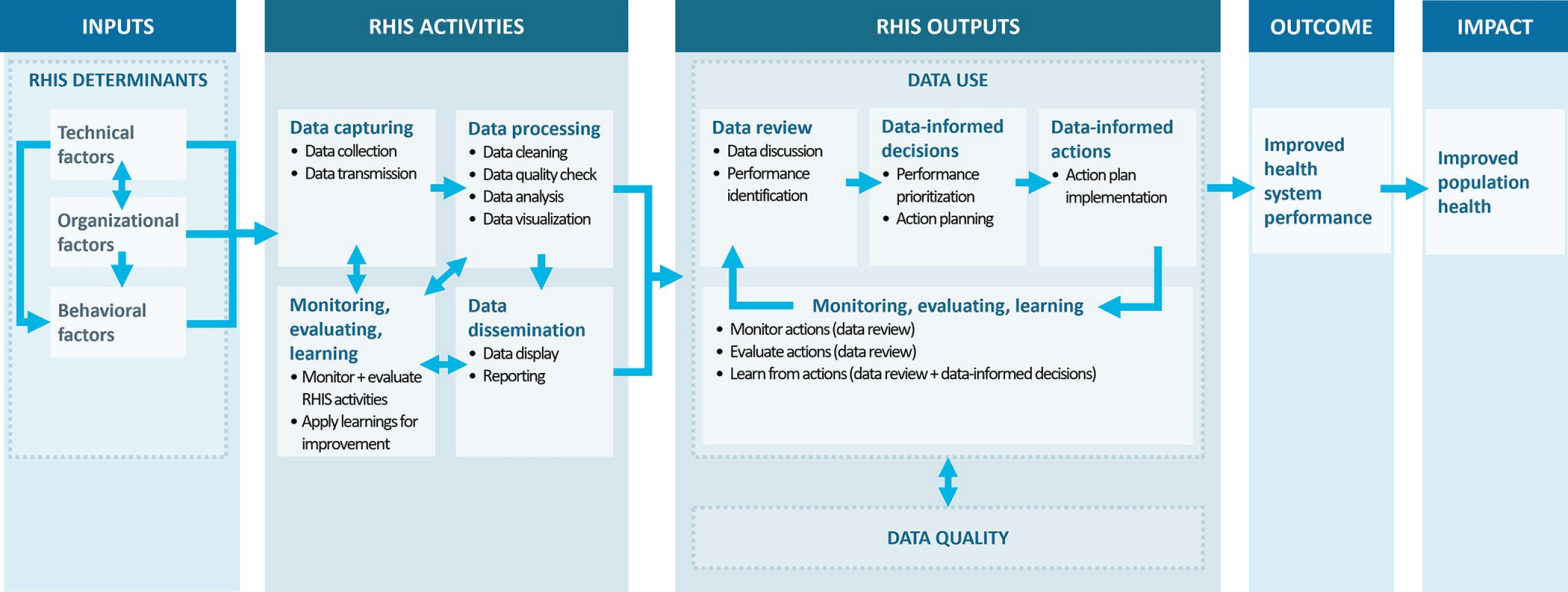
PRISM-Act framework.

We refined the PRISM framework, “PRISM-Act” (Fig 2), informed by the findings from this review.

The PRISM-Act framework maintains the original PRISM framework structure, in that the technical, organizational, and behavioral factors are determinants of RHIS data activities, which have RHIS outputs of data use and data quality, which contribute to improved health system performance, ultimately contributing to improved population health. The relationship between RHIS determinants is unchanged from the PRISM framework.

The PRISM-Act framework reflects two key changes to the PRISM framework. First, additional tasks have been added to the RHIS activities section and these tasks have been grouped into similar tasks of “data capturing,” “data processing,” “data dissemination,” and “monitoring, evaluating, learning.” The “data processing” and “data dissemination” boxes clarify that these RHIS data tasks are not a part of data use; instead, they are preceding steps to data use. The arrows in the framework indicate that data must be processed before it can be disseminated and used, though processed data can be used without dissemination of data via data display or reporting. These changes were made to address major conceptual and measurement issues identified in the literature. Despite general agreement among authors that data processing and dissemination are not a part of data use, many studies continue to include data analysis and data display as part of their data use measure. Strengthening data analysis capacity at the district and health facility-levels is important, but it is imperative to recognize that data analysis alone will not translate data to action nor lead to improvements to health system performance. Additionally, despite the frequency in which “reporting” is mentioned across the RHIS data use literature, this RHIS data task does not appear in the original PRISM framework. Given the issue of “mailbox syndrome,” where analyzed RHIS data is reported to higher levels but not used to inform decisions and actions at the health facility [21–24], and general agreement that reporting is not a part of data use, this step was added to the framework and presented as conceptually separate from data use. The "monitoring, evaluating, and learning” box and its arrows indicate the feedback cycle in which RHIS activities are monitored and evaluated and that learnings are applied to make improvements to RHIS activities such as approaches to RHIS data collection and analysis.

The second key difference is that in the PRISM-Act framework, “data use” has been conceptualized to be a multi-step process. Once data has been captured and processed, the data is ready to be reviewed. During the review, discussions take place to identify performance strengths and gaps informed by RHIS data; decision making is not a central focus of this step. Data review informs decisions regarding district or facility priorities and action steps. The action planning process includes proposing, considering, and selecting actions that address identified priorities, informed by RHIS data. This is then followed by a step that focuses on acting on these decisions; again, decision making is not a central focus of this step. This is the critical step where data is translated into action, and it is these actions that ultimately lead to improvements in health system performance that increase access to quality care. Actions are then monitored and evaluated to assess action progress and impact, informed by RHIS data, and the learnings from this step inform the start of another cycle of RHIS data review, decisions, and actions. The key difference between the “monitoring, evaluating, and learning” (MEL) boxes under “RHIS activities” and “RHIS outputs” is their purpose. That is, the MEL box under “RHIS activities” is closely tied to data quality checks in that its purpose is to improve RHIS activities in order to improve data quality and facilitate data use (i.e., the output of RHIS activities). The purpose of MEL under “RHIS outputs–data use” is to monitor, evaluate, and improve RHIS data use in order to contribute to improved health system performance (i.e., the outcome of RHIS data use).

By incorporating the data use terminology, definitions, and steps identified during the synthesis of the literature, the refined PRISM-Act framework aims to answer the questions, “What is RHIS data use and what is its relationship to decision making? What does RHIS data use look like? How do we know that data has been ‘used’?”

### Proposed RHIS data use definition

Findings from this review informed the development of the definitions for RHIS data use and related terms used in the refined PRISM-Act framework (Fig 2). Definitions for these terms were primarily extracted from three articles which explicitly defined RHIS data use steps [12,17,46] and merged; these are presented in Table 8.

**Table 8:** Proposed RHIS data use terminology and definitions.

Terminology	Definition
**RHIS data use**	The process by which health staff review and discuss analyzed RHIS data and collectively identify, address, and monitor health system performance gaps and priorities informed by RHIS data.
**RHIS data review**	A step in the RHIS data use process, by which health staff review and discuss analyzed RHIS data and identify health system performance strengths and gaps informed by RHIS data, including analyses of the reasons for the gaps.
**RHIS data-informed decision**	A step in the RHIS data use process, by which health staff review analyzed RHIS data to reach a collective decision about priorities and action steps. Action planning includes proposing, considering, and selecting actions and making plans to implement the actions, such as identification of resources needed and responsible persons.
**RHIS data-informed action**	A step in the RHIS data use process, by which health staff implement action plans based on RHIS data-informed decisions.
**RHIS data-informed action plan monitoring, evaluation, and learning**	A step in the RHIS data use process, by which health staff monitor progress on the action plan, evaluate its impact on the priorities informed by RHIS data, and learn from the process and make adjustments as needed.

## Discussion

This review identified 45 papers investigating RHIS data use at the district or health facility-level in LMICs, of which 24 measured RHIS data use. More than a third of the 33 original research articles were from Ethiopia, which may be explained by Ethiopia’s commitment to an “information revolution,” with particular emphasis on enhancing RHIS data use at the local administrative levels [17,38,45,46,54].

Text analysis of selected studies revealed that there is a lack of consensus and consistency in using terms to describe “RHIS data use” and studies often do not provide a definition for “RHIS data use.” This is consistent with recent systematic review findings of a need for more clarity and consensus in conceptualizing and measuring data use [15,28]. There were also differences in whether to define data use by its application or by the steps it encompasses. Data use definitions more commonly answered the question, “For what purposes can the data be used?” and were less likely to answer the questions, “What does data use look like? How do we know that data has been ‘used’?” Defining RHIS data use by its application alone is defining RHIS data use by its intended outcome rather than defining what it actually is. Using logic model terminology [63], it is insufficient and incorrect to define the output, “RHIS data use” by the outcome, “improved health system performance.” Future studies should ensure that RHIS data use is defined by what it encompasses, rather than by its application alone.

Both data use terminology and definitions used in the literature indicate that data use is strongly connected with decision making, but there lacked clarity and consistency about whether data use was defined by decision making. In other words, is data considered “used” if review and discussion of analyzed data does not lead to a decision? There was also some inconsistency in language and measurement approach in considering if RHIS data use is inclusive or exclusive of data tasks such as data analysis, reporting, and data interpretation, though more authors considered these tasks to precede, and not be a part of, data use. Similarly, there was some disagreement about whether data review and discussion were a part of data use, but more authors viewed these to be a part of data use, along with problem identification, prioritization, decision making, action planning, action, and monitoring.

The refined PRISM-Act framework aims to address these issues by outlining which RHIS data tasks, or steps, precede or are a part of “data use.” Separating RHIS data use into specific steps also highlights the role of RHIS data in each of these steps, when decision making occurs, and that action is a necessary step for any improvements to health system performance. This approach also better accommodates the reality that data is only one part of successful decision making and action [12,64], and the level of influence of the determinants of data use likely differ across each data use step.

### Recommendations for improving data use measurement approaches

Based on the findings from this review, four key recommendations were identified to improve approaches to measuring RHIS data use: (1) clearly delineate RHIS data tasks, (2) use objective data collection approaches to measure RHIS data use, (3) select the appropriate RHIS data use outcome variable type, and (4) comprehensively report RHIS data use measurement approaches.

First, studies should clearly delineate RHIS data tasks and measure these activities separately. Many studies continue to include data analysis and data visualization activities in their measurement of data use, despite consensus that these activities precede, and are not a part of, data use. Use of the refined PRISM-Act framework should address this issue, by ensuring that each delineated RHIS data task, such as data processing and data analysis, are measured separately. A 2020 study from Ethiopia [17] made an important discovery when disaggregating data use into multiple steps and measuring each step separately. While a low proportion of health facilities reviewed their performance monthly (performance identification), an even lower proportion prepared action plans after reviewing performance (prioritization and action planning). Similarly, “mailbox syndrome” was observed in South Africa and Tanzania, where facility-level data were analyzed for reporting to higher levels, but not reviewed and discussed to inform facility-level decisions and actions [23,24]. This highlights the importance of measuring data analysis, reporting, and data-informed decisions and actions separately, which will help identify which RHIS data tasks are functioning well, and which require additional support. Endriyas et al.’s [17] approach to measuring data use steps can serve as a helpful resource for implementers and researchers.

Secondly, studies should aim to use more objective data collection methods such as observation and document review when collecting RHIS data use measurements. Many studies using the adapted version of the PRISM toolkit surveyed district and health facility staff to collect self-reported measures of data use, which are subject to social desirability and recall bias [34]. This adaptation diminishes the validity of the PRISM toolkit, which intentionally selected record observation as a gold standard data collection approach [9]. A study that measured data use through both self-reported and observed measures, found that participants reported higher levels of data use than what was observed through review of action plan implementation reports [17]. Endriyas [17], Prakash [51], and Wude [42] have used more objective measurement approaches and can serve as examples for future studies.

Third, the appropriate outcome variable type should be selected when conducting hypothesis testing and tests of association with data use as the outcome. Almost all studies testing the association of data use with potential determinants transformed a continuous data use variable into a binary “good” or “poor” data use variable, using the mean or median value as the cut-off. There are several disadvantages of dichotomizing a continuous outcome. First, substantial information is lost, such that the statistical power to detect an association between the outcome and predictor is reduced [65]. Dichotomizing a continuous variable at the median has been found to be equivalent to discarding a third of the data [65]. Additionally, using a cut-off to create a binary variable, such as “good” or “poor” data use oversimplifies the data. Data points near the cut-off are dichotomized to good or poor despite being very similar on the continuous scale, which can lead to incorrect conclusions. Use of a cut-off has been shown to increase the risk of spuriously significant results by both overestimating the difference between groups and narrowing its confidence interval [65]. Furthermore, variation of cut-off values across studies can make comparison and meta-analysis challenging [66]. If data use is collected and calculated as a continuous variable, it is beneficial to maintain it as a continuous variable for tests of association. Binary measures of data use should only be used when the outcome is a specific, “yes, data was used in this event,” such as whether a singular decision at a health facility was made based on RHIS data.

Lastly, studies measuring RHIS data use should comprehensively and clearly report their data use measurement approaches. This should include the type of data collection tool used (e.g., document review, questionnaire), a list of each item on the tool and its response options (e.g., yes/no; five-point agreement Likert scale), and how the data use composite measure is calculated based on these items. If possible, a supplementary file of the data collection tool should be included. Dagnew [41], Endriyas [17], Odei-Lartey [57], Prakash [51], and Seid [44] are examples of studies comprehensively reporting their data use measurement approach. Clear reporting of methods will support researchers to advance RHIS data use measurement approaches.

### Recommendations for future research

There remain several gaps in the literature on RHIS data use at the district and facility levels. Overall, there is a need for more quantitative studies to better understand the level and determinants of RHIS data use across different resource limited settings. Very little quantitative or qualitative exploration has been done to understand the level and determinants of data-informed decisions and actions, nor regarding the relationship between data-informed decisions and actions. In other words, what aspects of data-informed decisions successfully lead to action? There is also a paucity of studies that report on actual decisions made and actions taken by health facilities based on RHIS data, as well as studies that have evaluated the impact of these decisions and actions on health system performance. This type of evidence is essential in informing the development of more effective RHIS data use interventions that support the translation of data into action.

### Limitations

This review has some limitations. The literature search was limited to published peer-reviewed articles in English and thus is not representative of materials available in other languages and sources such as the grey literature. This may have narrowed the boundaries of the exploration of how RHIS data use is defined in the literature but aided in maintaining a level of methodological quality for the exploration of how RHIS data use is conceptualized and measured. The database searches were limited to four databases, but the high number of duplicate records identified through this search strategy and the relatively small number of articles identified from citation mining indicates the comprehensiveness of this review’s search strategy.

Another limitation is that, while the RHIS data use steps in the PRISM-Act framework were developed informed by the literature which used both quantitative and qualitative methods, the framework itself was not validated through qualitative and quantitative methods. Future qualitative studies should explore if key RHIS data use steps are represented in the PRISM-Act and describe the importance of each step; quantitative studies should measure RHIS data tasks and assess their association with the subsequent steps, including the association with RHIS data use steps and the outcome, improved health system performance. These findings from future studies may inform further refinements to the PRISM-Act framework.

Given the rarity of studies that measured RHIS data use steps and assessed its determinants, the refined framework assumed that these relationships remained the same as was proposed in the original PRISM framework. Similarly, this review did not explore the relationship between RHIS data use steps and RHIS data quality and maintained the same relationship proposed in the original PRISM framework. Future research is needed to better understand these relationships.

### Conclusions

This review fills a critical gap in the RHIS data use literature by developing a more refined definition and framework for RHIS data use and proposing improved approaches to measuring RHIS data use. Despite recognition of the critical importance of transforming data to action, RHIS data use interventions and studies have more often focused on RHIS activities such as data collection and analysis rather than on data-informed decisions and actions. The refined PRISM-Act framework illustrates that RHIS data use is a multi-step process that includes data-informed decisions and actions, and that it is in the implementation of actions that the performance of the health system improves. Future studies exploring the determinants and relationships between these RHIS data use steps will help expand the evidence-base to inform the design of more targeted and effective RHIS strengthening strategies that ultimately lead to improved health system performance.

## Supporting information

S1 File PRISMA 2020 checklist. (DOCX)

S1 Table Study eligibility criteria. (DOCX)

S2 Table Study search terms. (DOCX)
