# Supplementary material for: Refining the Performance of Routine Information System Management (PRISM) framework for data use at the local level: An integrative review
Source: PLoS One. 2023 Jun 27;18(6):e0287635. doi: 10.1371/journal.pone.0287635 (PMC10298795; doi:10.1371/journal.pone.0287635)
Supplement: S1 Table — (DOCX) [file pone.0287635.s002.docx]

S2 Table. Study eligibility criteria

| **Inclusion criteria** | **Exclusion criteria** |
| --- | --- |
| Published in peer-reviewed scientific journals | Grey literature |
| Studies of any design (quantitative, qualitative, mixed methods, synthesis) investigating data use at the district or health facility-level | Studies not focusing on RHIS data use  Studies of RHIS data use at the global, national, and/or regional-levels  Research protocols, editorials, description of interventions |
| Studies conducted in LMICs according to the Development Assistance Committee list [65] | Studies conducted in high-income countries |
| Studies published in English | Studies in languages other than English |
| Studies published from January 2009 to December 2021 | Studies published before January 2009 |
