# Supplementary material for: Refining the Performance of Routine Information System Management (PRISM) framework for data use at the local level: An integrative review
Source: PLoS One. 2023 Jun 27;18(6):e0287635. doi: 10.1371/journal.pone.0287635 (PMC10298795; doi:10.1371/journal.pone.0287635)
Supplement: S2 Table — (DOCX) [file pone.0287635.s003.docx]

S3 Table. Study search terms

| **First term** |  | **Second term** |
| --- | --- | --- |
| Routine health information system or  health management information system | and | Data use or data utilization or  Information use or information utilization or  Data-informed or data-driven or  Decision making or  Quality improvement or  Data to action or  Audit and feedback |
| Data use or data utilization or  Information use or information utilization or  Data-informed or data-driven or  Decision making or  Quality improvement or  Data to action or  Audit and feedback | and | Measure |
| Performance of Routine Information System Management |  |  |
